# Effects of Qinghuang Powder on Acute Myeloid Leukemia Based on Network Pharmacology, Molecular Docking, and In Vitro Experiments

**DOI:** 10.1155/2021/6195174

**Published:** 2021-12-28

**Authors:** Ying-jian Zeng, Min Wu, Huan Zhang, Xin-ping Wu, Lu Zhou, Na Wan, Zhen-hui Wu

**Affiliations:** ^1^Jiangxi University of Chinese Medicine, Nanchang 330004, Jiangxi Province, China; ^2^The Affiliated Hospital of Jiangxi University of Chinese Medicine, Nanchang 330006, Jiangxi Province, China

## Abstract

Qinghuang powder (QHP) is a traditional Chinese herbal medicine. This is a unique formula that is frequently used to treat malignant hematological diseases such as acute myeloid leukemia (AML) in modern clinical practice. An approach of network pharmacology and experimental validation were applied to investigate the pharmacological mechanisms of QHP in AML treatment. First, public databases for target genes known to be associated with AML are searched and compared to the target genes of the active compounds in QHP. Second, AML-associated genes and QHP target genes are compared to identify overlapping enriched genes, and these were used to predict selected target genes that may be implicated in the effects of QHP on AML. Additionally, we conducted functional enrichment analyses, such as gene ontology (GO) and the Kyoto Encyclopedia of Genes and Genomes (KEGG) pathways. The significantly enriched pathway associated with potential target proteins was the PI3K-Akt signaling pathway, suggesting that these potential target proteins and pathways may mediate the beneficial biological effects of QHP on AML. All these following genes were found to occur in the compounds-target-pathway networks: AKT1, MAPK1, MAPK3, PIK3CG, CASP3, CASP9, TNF, TGFB1, MAPK8, and TP53. Then, based on the molecular docking studies, it was suggested that the active compound isovitexin can fit into the binding pockets of the top candidate QHP-AML target proteins (PIK3CG). Subsequently, based on the prediction by network pharmacology analysis, both in vitro AML cells and western blot experiments were performed to validate the curative role of QHP. QHP exerted its antitumor activity on AML in vitro, as it inhibits cells proliferation, reduced the expression of Bcl-2 protein, and downregulated the PI3K-Akt signaling pathway. In conclusion, these results revealed that QHP could treat AML via a “multicomponent, multitarget, multipathway” regulatory network. Furthermore, our study also demonstrated that the combination of network pharmacology with the experimental study is effective in discovering and identifying QHP in the treatment of AML and its underlying pharmacological mechanisms.

## 1. Introduction

Acute myeloid leukemia (AML) is an aggressive malignancy worldwide with a poor prognosis. It is a relatively rare type of cancer (statistically accounting for 1.1% of all new cancer cases), with an estimated 19, 940 new cases in the United States in 2020, according to the NIH (National Institutes of Health) and SEER (Surveillance, Epidemiology, and End Results) databases. AML is characterized by the rapid proliferation of immature myeloid leukemia cells [1]. Most patients suffering from AML are struggling for life every day because it has the lowest overall survival rate of all cancer, in spite of aggressive treatments with chemotherapy. One of the biggest obstacles in AML treatment is the high relapsing rate despite a positive response to chemotherapy. Many drugs are used to treat AML, including FLT3 inhibitors (midostaurin), IDH inhibitors (ivosidenib and enasidenib), hedgehog pathway inhibitors (glasdegib), Bcl-2 inhibitors (venetoclax), and proteasome inhibitors (bortezomib). However, the action of these drugs can suppress the progression of AML and is associated with several adverse side effects including neutropenic fever, infections, hyperleucocytosis, and gastrointestinal symptoms [2, 3].

Evolving pharmacological treatment strategies have led to the development of more comprehensive and multistage AML therapies, and patients are now able to use complementary and alternative medicine therapies. Traditional Chinese medicine (TCM) is a commonly used alternative medicine therapy for AML patients [4–12]. TCM has been used widely in China for more than 2,000 years and has gradually gained international recognition due to its outstanding efficacy. For example, the exceptional Chinese scientist Tu Youyou found that artemisinin, a substance extracted from a medicinal plant that was described in the third-century book Emergency Prescriptions to Keep up Your Sleeve (Zhouhou Beiji Fang) as a treatment for malaria, could be developed into a pharmaceutical drug. The work of Dr. Tu and her team eventually led to an effective treatment that saved the lives of millions of people. She along with two other scientists was awarded the Nobel Prize for their research.

Qinghuang powder (QHP, Realgar-Indigo Naturalis formula) was used as a folk Chinese medicine, and this medicine is also described in many ancient medical books, such as Jing Yue Quan Shu, Shi Yi De Xiao Fang, and Qi Xiao Liang Fang. QHP is composed of the Chinese herbs Qing Dai (Indigo Naturalis) and Xiong Huang (Realgar). The compounds of Indigo Naturalis include indigo, indirubin, proteins, tannic acid, and inorganic salts. The constituents of Realgar include arsenic disulfide (As_2_S_2_), with a small amount of arsenic trioxide (As_2_O_3_) and other heavy metal salts. QHP has been used to treat malignant hematological diseases including AML, chronic myeloid leukemia (CML), and myelodysplastic syndrome [13–17]. However, very little research on the mechanism underlying the therapeutic effects of QHP in AML has been conducted.

Network pharmacology-based drug discovery is an emerging, cost-effective drug development approach incorporating systems biology, bioinformatics, and poly-pharmacology [18]. Network pharmacology utilizes network structures to discover and analyze multicomponent and multitarget drugs [19, 20]. Chinese herbal medicines such as QHP are potential “drugs” that could be the basis for developing multicomponent and multitarget synergistic AML therapeutics.

In this study, we examined the active components and mechanisms underlying the effects of QHP on AML using network pharmacology analysis in combination with molecular docking and experimental validation. This study was designed using Network Pharmacology Evaluation Method Guidance-Draft [21].

## 2. Materials and Methods

### 2.1. Network Pharmacology

#### 2.1.1. Chemical Components Screening

All components of QHP were retrieved from the Symptom Mapping Database [22] (SymMap; http://www.symmap.org/), TCM Systematic Pharmacology Database [23] (TCMSP; https://tcmspw.com/index.php), and TCM Integrated Database [24] (TCMID; http://www.megabionet.org/tcmid/) and supplemented by literature mining. The oral administration of TCM therapeutics must overcome the barriers which come in the form of absorption, distribution, metabolism, and excretion (ADME) processes to be active [25]. In ADME processes, oral bioavailability (OB) and drug-likeness (DL) are the two most important parameters to measure the pharmacokinetic process of drugs in vivo. A good OB for a new drug candidate is of the most essential pharmacokinetic parameters. DL refers to physical and chemical properties such as stability, solubility, and biological properties. High OB is usually a crucial indicator for determining the DL index of active compounds. In the Drug Bank database, the average DL index is 0.18. The compounds with DL index ≥ 0.18 and OB ≥ 30% were regarded as better drugs and were better for the use of humankind [25]. The components meeting the OB ≥ 30% and DL ≥ 0.18 criteria were selected as active compounds in this study.

#### 2.1.2. Establishment of Target Library

The proteins and gene targets of the active compounds and AML-related human genes were collected from multiple databases. Information on target proteins or genes of active compounds was collected from the TCMSP, TCMID, and SymMap databases. For comprehensiveness, we used the Swiss Target Prediction database [26] (https://www.swisstargetprediction.ch/) and TargetNet [27] (https://targetnet.scbdd.com/calcnet/index/) to look for targets for the components Indigo Naturalis and Realgar. These active compounds were uploaded to an online database, the species was restricted to Homo sapiens, and the target data with probability > 0 were downloaded.

AML-related human genes were collected from several databases, namely, DrugBank (https://go.drugbank.com/), DisGeNET (https://www.disgenet.org/search), GeneCards (https://www.genecards.org/), and OMIM (https://omim.org/). All protein target names were identified and converted to names of standard genes via UniProt (https://www.uniprot.org/) [28]. After these were determined, the gene targets of QHP were combined, and repetitive targets were deleted. Similarly, the gene targets of AML from four databases were combined, and the repetitive targets were deleted. A Venn diagram of QHP-AML targets was plotted and visualized with web tools (https://bioinformatics.psb.ugent.be/webtools/Venn/). Finally, the QHP and AML targets that overlapped were collected using R software, and the intersection of overlapped targets was selected as the target library of this study.

#### 2.1.3. Bioinformatic Annotation

The gene ontology (GO) and Kyoto Encyclopedia of Genes and Genomes (KEGG) pathway enrichment analyses were conducted using the R software (version 3.6.0 for Windows). The GO enrichment analyses include biological process (BP), cellular component (CC), and molecular function (MF). The GO enrichment analysis for AML-related targets of QHP revealed remarkably 10 rich terms in BP, CC, and MF. The KEGG pathway enrichment analysis was used to elucidate the potential mechanism of QHP for AML. The bar charts and bubble diagrams were drawn with R-studio software (version 3.6.5 for Windows).

#### 2.1.4. Protein-Protein Interaction

Biological processes involve interaction between multiple body systems are regulated by a complex regulatory network, rather than just a single gene or protein. These interactions include direct (physical) and indirect (functional) associations. STRING (https://string-db.org/) is a database of known and predicted protein-protein interactions [29]. The target proteins were uploaded to the STRING online database, and the organism selected was limited to Homo sapiens sources. We then downloaded the protein-protein interaction (PPI) relationships that we discovered into TSV format and imported the TSV file into Cytoscape software (ver. 3.7.1) to construct the PPI network diagram. In this diagram, each node represents a protein, respectively, and the connection between nodes indicates an interaction between two proteins.

#### 2.1.5. Network Construction

Network construction was achieved with Cytoscape software (ver. 3.7.1). Cytoscape is an open-source software platform for visualizing molecular interaction networks and biological pathways and then integrating these networks with annotations, gene expression profiles, and other data [30]. Based on our previous work, we identified and visualized the compounds-targets-pathways (C-T-P) relationship between QHP and AML. This included 2 networks, named as follows: (1) the active compounds-potential targets network of QHP for targeting AML; (2) active compounds-potential targets-pathways network of QHP for targeting AML.

### 2.2. Molecular Docking

To further validate the effects of QHP acting on AML, molecular docking technology was applied for screening the active compounds and candidate proteins. Molecular docking technology is an important method for the screening of drugs, which starts from known proteins and small-molecule compounds and identifies them by simulating the geometry and energy matching. We used the RCSB protein data bank (https://www.rcsb.org/) database to retrieve related protein information and downloaded the 3D structure files of the candidate proteins as the receptors. We then modified them using PyMol software (version 1.8) and AutoDockTools software (version 1.5.6) to isolate the original ligands, remove water molecules, add hydrogen, and patch amino acids. The mol2 structure of the active compounds was set to rotatable and saved as a “pdbqt” format file through the Autodock Tools software. Finally, we performed molecular docking using Autodock-vina software (version 1.1.2). The docking results were visualized with PyMol software, and the docking interaction patterns were established.

### 2.3. Experiment Verification

#### 2.3.1. Reagents and Drugs

Isovitexin (lot no. abs47023192) was obtained from Absin Bioscience Inc. Roswell Park Memorial Institute (RPMI) 1640 (lot no. RHBH8770) and DMSO (lot no. RNBD8012) were purchased from Sigma-Aldrich (Shanghai) Trading Co., Ltd. FBS (lot no. 42F7180 K) was purchased from Gibco Life Technologies Ltd. Cell Counting Kit-8 (CCK-8) kit (lot no. KGA317) was obtained from KeyGEN Biotech Co., Ltd. RIPA lysis buffer (lot no. P0013 B), PMSF (lot no. 020421210524), BeyoECL kit (lot no. P0018AS), SDS-PAGE Sample Loading Buffer 5 × (lot no. 070121210811), and BCA protein quantitation kit (Lot No. 062521210726) were purchased from Beyotime Institute of Biotechnology. BSA (lot no. EZ4567D106) was purchased from Guangzhou Saiguo Biotech Co., Ltd. Anti-PIK3CG (lot no. 5405S) were purchased from Cell Signaling Technology, Inc. Anti-AKT (lot no. CJ38131), anti-Bcl-2 (lot no. CN48171), and goat anti-mouse IgG (lot no. BS12478) were purchased from Bioworld Technology, Inc. Anti-GAPDH (lot no. AB0037) was purchased from Nanjing JinZai Biotechnology Co., Ltd. Anti-Rabbit IgG (lot no. 147832) was purchased from Jackson ImmunoResearch Laboratories, Inc.

#### 2.3.2. Cell Source and Culture

KG1-a cells were gifted from the Southern Medical University. HL-60 cells were purchased from JiangSu Rongtai Biotech Co., Ltd. Both KG1-a cells and HL-60 cells were cultured in RPMI-1640 containing 10% FBS and 1% penicillin-streptomycin at 37 °C in an incubator containing 5% CO_2_. Once cell confluence reached∼80%, these cells were passaged.

#### 2.3.3. Cell Viability Assay

The viability of the KG1-a cells and HL-60 cells was determined using CCK-8 kit. Firstly, KG1-a cells and HL-60 cells were suspended, respectively, in RPMI-1640 (5 × 103 cells/well in 100 *μ*L) and sown in Corning disposable 96-well plates for 24 h. Different concentrations of isovitexin (in 0.024, 0.098, 0.39, 1.56, 6.25, 25, and 100 *μ*mol/L range) were incubated with KG1-a cells and HL-60 cells for 48 h. Then, the culture medium of 96-well plates was added with CCK-8 solution (culture medium: CCK-8 solution = 10 : 1). Lastly, 96-well plates were incubated at 37 °C for 1.5 h. The absorbance was read at 450 nm using Multiskan MK3 microplate reader (Thermo Fisher Scientific, Inc.). The half-maximal inhibitory concentration (IC50) of isovitexin was calculated using GraphPad Prism 8.3.0 software.

#### 2.3.4. Western Blot Analysis

Further to study how isovitexin affects these cell signaling pathways, KG1-a cells and HL-60 cells were plated in 6-well plates (1 × 104 cell/well) and treated for 48 h with a variety of isovitexin concentrations (0.39, 1.56, 6.25, and 25 *μ*mol/L). These cells were collected from the 6-well plates followed by the addition of RIPA lysis buffer (containing 1 mM PMSF) and incubated on ice for 15 min. The recovered lysate was centrifuged at 4 °C at 14,000 ×g for 10 min. Thereafter, the protein concentration was measured with a BCA Protein Assay Kit. An equal amount of protein (50 *μ*g/lane) was separated by 12 SDS-PAGE and electroblotted onto polyvinylidene difluoride (PVDF) membranes (Millipore). Subsequently, the PVDF membranes were blocked with 5% skim milk for 1 h and incubated overnight with different primary antibodies against PIK3CG (1 : 1,000 dilution), AKT (1 : 500 dilution), Bcl-2 (1 : 500 dilution), and GAPDH (1 : 3,000 dilution), respectively, at 4 °C. Following 3 washes, the membranes were incubated for 1 h with secondary horseradish peroxidase-conjugated antibody at room temperature. Immunolabeled protein bands were detected with ECL substrate. Semiquantitative analysis was performed using ImageJ software. Target protein levels were normalized against the level of GAPDH.

### 2.4. Statistical Analysis

Data were expressed as mean and standard deviation (SD) from at least three independent experiments. The statistical significance of differences between the two groups was determined using Student's *t*-test or LSD test. For multiple comparisons, one-way ANOVA was performed with SPSS software (version 22.0; IBM Corp., Armonk, NY, USA). Statistical significance was set at *p* value < 0.05.

## 3. Results

### 3.1. Active Compounds in QHP

The QHP formula contains two Chinese medicinal constituents. They are Indigo Naturalis and Realgar. We identified a total of 48 compounds in QHP through the TCMSP, TCMID, and SymMap databases, 38 of which were contained in Indigo Naturalis and 10 in Realgar. Among the 38 compounds in Indigo Naturalis, 8 (26.3%) met the requirement of OB ≥ 30% and DL ≥ 0.18 based on the TCMSP database, and the 4 compounds in Realgar also met this criterion based on the TCMID and SymMap databases. Therefore, 14 compounds were chosen as candidate active compounds for further analysis. A detailed list of these compounds is shown in Table 1. Through this process, we found that four of these compounds (As_2_O_3_, As, AsS, and As_4_S_4_) do not meet the ADME parameters and were therefore excluded.

### 3.2. Targets Identification of QHP on AML

**Figure 1:**
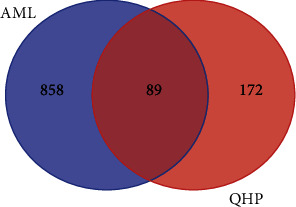
Venn diagram of QHP-AML intersection targets.

Among the 14 active compounds, 319 target genes were retrieved from the TCMSP, TCMID, TargetNet, and SymMap databases. After eliminating duplicates, 261 gene targets were obtained for further study. In total, 947 AML-related human gene targets were collected from OMIM, GeneCards, DisGenet, DrugBank, and SymMap databases. We then extracted all the unique gene targets from each result. The intersection of these two types of gene targets was then collected for further analysis (Figure 1). As a result, we found that 89 gene targets from 13 compounds (bisindigotin was removed as it has no related target genes) in QHP were associated with AML.

### 3.3. Compounds-Target Network and Analysis

**Figure 2:**
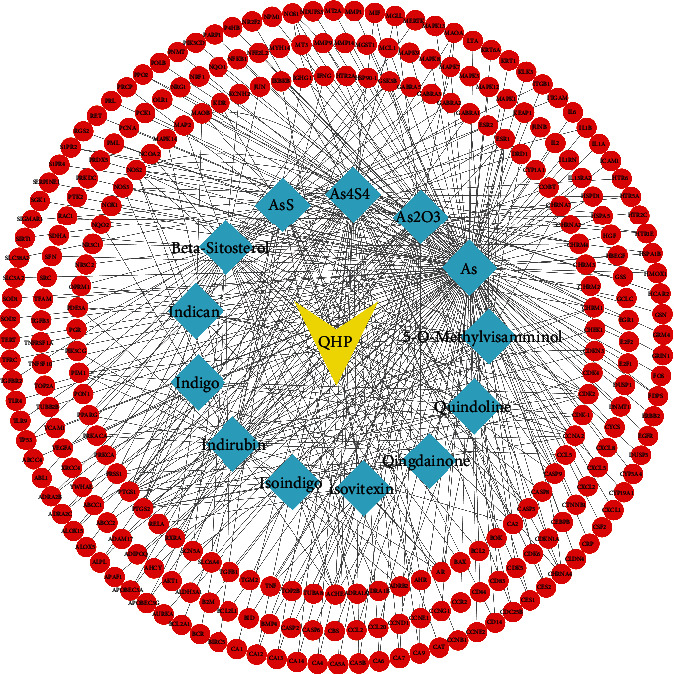
The compounds-target network for QHP on AML. The red node represents the target point, the yellow node represents the Chinese herbal medicine formula point, and the cyan node represents compound points.

The targets of 13 compounds that were associated with AML were imported into Cytoscape (ver. 3.7.1), and the QHP-AML target network diagram that was constructed is shown in Figure 2. Our analysis of the QHP-AML target network showed that it contains 274 nodes and 434 edges. The yellow node in Figure 2 represents the Chinese herbal medicine formula point, the cyan node represents compound points, and the red node represents target points. These bioactive compounds were associated with multiple AML targets, namely, As (degree = 147), As_4_S_4_ (degree = 60), beta-sitosterol (MOL000358, degree = 52), As_2_O_3_ (degree = 46), indirubin (MOL002309, degree = 36), AsS (degree = 23), 5-O-methylvisamminol (MOL001753, degree = 21), indican (MOL011105, degree = 12), indigo (MOL001781, degree = 9), isoindigo (MOL011335, degree = 8), isovitexin (MOL002322, degree = 7), qingdainone (MOL001810, degree = 7), and quindoline (MOL011332, degree = 6). These high-degree compounds in the network were deemed to relate to the essential therapeutic effects of QHP on AML.

### 3.4. Network of Protein-Protein Interaction

**Figure 3:**
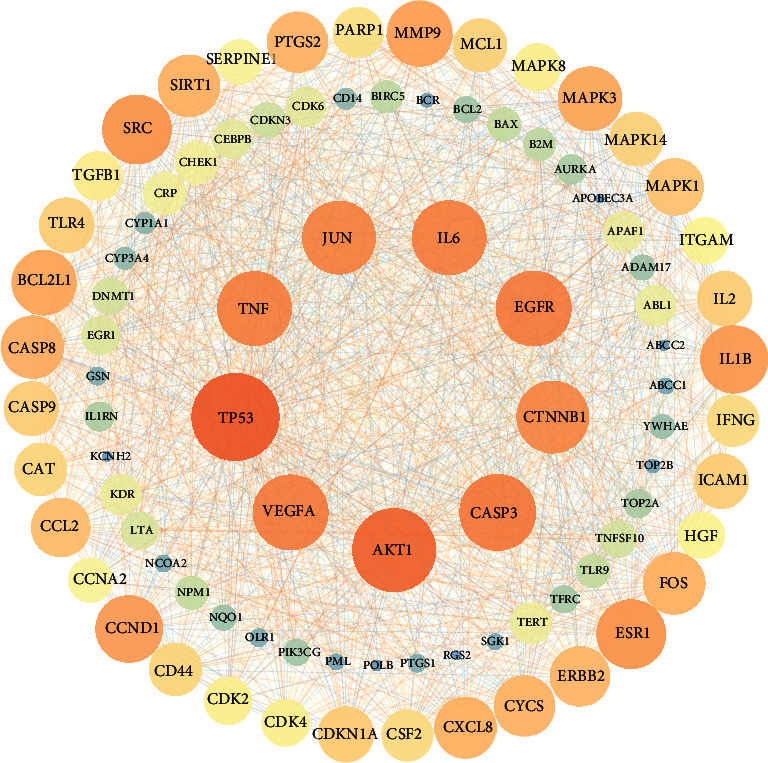
The node size and color shade are positively correlated with the degree.

The 89 gene targets shared between QHP and AML were imported into the STRING database (https://www.string-db.org/), and the results were downloaded as a “TSV” file. The TSV file was imported into Cytoscape (ver. 3.7.1), and the diagram of the gene target network was constructed as shown in Figure 3. By analyzing the gene target network, we found that the network includes 89 nodes and 1568 edges, and the degrees represent the number of connected edges (the edges represent relations between two adjacent nodes) of nodes. The average node degree was 35.24, and the average clustering coefficient was 0.74. The higher the degree, the greater the regulatory role of nodes plays in the network. We selected those genes with an above-average node degree. As a result, only 9 targets had good network connectivity characteristics. These 9 targets that may be core genes are shown in Table 2.

### 3.5. Networks and Enriched Functions in AML-Associated Genes

#### 3.5.1. GO and KEGG

**Figure 4:**
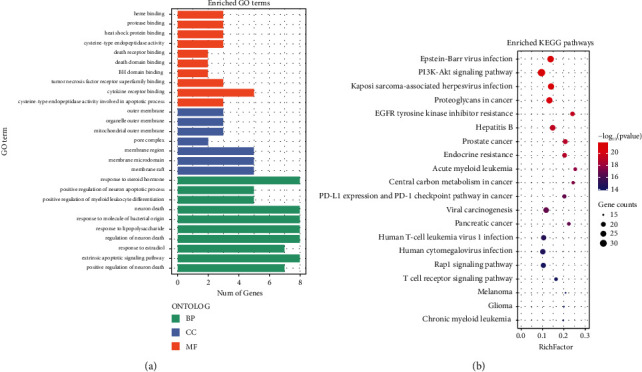
The top 10 significance of enriched GO terms (a) and top 20 significance of enriched KEGG pathways (b) analysis of therapy target genes of QHP on AML.

In order to further identify the functional characteristics of putative target genes of QHP on AML in detail, the GO and KEGG pathways enrichment analyses of target genes were performed using the R statistical language. GO enrichment analysis showed that these genes were involved in three aspects of biological functions: biological process (BP), cellular component (CC), and molecular function (MF). The top 10 significantly enriched GO terms in BP, CC, and MF of the potential targets are shown in Figure 4(a). The GO enrichment analysis indicated that the targets of QHP were correlated with the BP of response to molecule of bacterial origin, response to lipopolysaccharide, extrinsic apoptotic signaling pathway, and other processes. The CCs include membrane raft, membrane microdomain, and membrane region. These targets of QHP are also involved in the MF, including cytokine receptor binding, protein serine/threonine kinase activity, and receptor ligand activity. To analyze the underlying KEGG pathways of QHP that may be responsible for its effects on AML, pathway enrichment analysis was conducted. The top 20 significantly enriched KEGG pathways are shown in Figure 4(b). The pathway of PI3K-Akt signaling pathway exhibited the largest number of related gene targets (34 counts).

#### 3.5.2. Compounds-Target-Pathway Network Analysis

**Figure 5:**
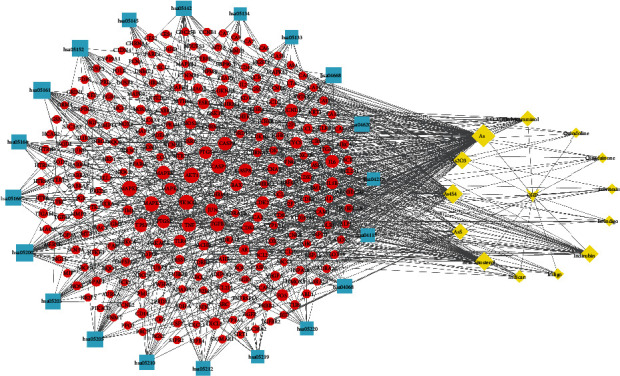
The compounds-target-pathway network. The cyan node represents pathways; the red node represents targets, and the yellow node represents compounds. The edges represent the interactions between compounds, target, and pathway, and node size is proportional to their degree.

Based on the above GO terms and KEGG pathway enrichment analyses, the compounds-target-pathway network was generated with Cytoscape (version 3.7.2), which connected these compounds, potential targets, and corresponding pathways. The compounds-target-pathway network is shown in Figure 5; it includes 173 nodes (1 Chinese herbal medicine formula node, 13 active compounds nodes, 260 potential target nodes, and 20 pathway nodes) and 799 edges. The yellow diamonds represent compounds, red circles correspond to targets, and cyan rectangles represent pathways.

### 3.6. Molecular Docking

**Figure 6:**
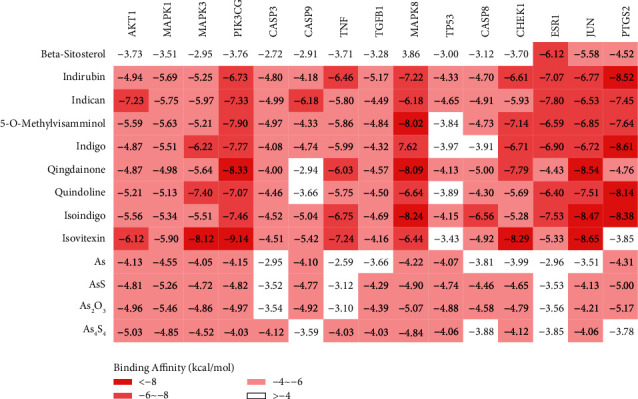
Heat map of the respective lowest binding energy value distribution of key targets and related components by molecular docking.

In order to further validate the ability of the active compounds in QHP to bind with the key targets, molecular docking visualization techniques were used to examine systematic docking. We chose to research these underlying target proteins in the C-T-P network because they were high-degree nodes with multifunctional connections. The top 10 key targets based on degree were as follows: AKT1, MAPK1, MAPK3, PIK3CG, CASP3, CASP9, TNF, TGFB1, MAPK8, and TP53. A total of 13 compounds were selected for docking on the 10 target proteins under the procedure. The thermal map of the lowest binding energy is shown in Figure 6.

**Figure 7:**
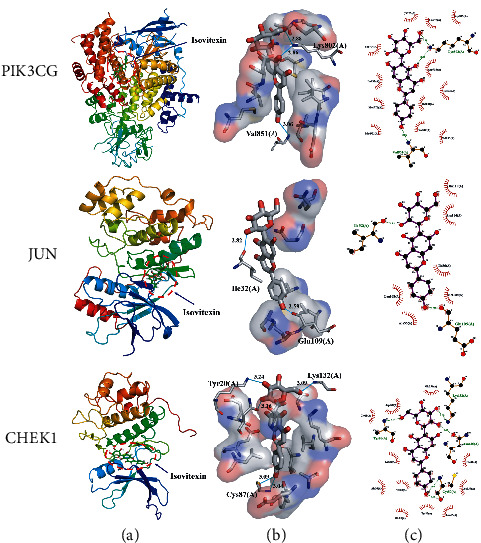
The binding pattern of the key targets of the network and the lowest component of their binding affinity. (a) Overall view of docking, with proteins represented by cartoon models; (b) three-dimensional detail of the docking interaction, with the small molecule represented by green sticks; (c) two-dimensional detailed view of the hydrophobic interactions between small molecules and protein residues.

Docking analysis successfully predicted docking between QHP and the binding pockets of 10 target proteins. The most active compound in QHP based on docking results was isovitexin. The value of binding affinity (kcal/mol^−1^) was below −7.0, indicating good binding activity between the compound and the protein [31]. The lower the binding affinity value, the better the docking effect. Isovitexin bounds to PIK3CG via hydrogen bonds with Val851 (3.06 Å) and Lys802 (2.86 Å, 2.88 Å) and had multiple hydrophobic interactions with residues Asp805, Ala775, Lys776, Ser774, Ile932, Met772, Met922, Val850, Glu849, Ile800, Ile848, and Asp933. Similarly, isovitexin was predicted to dock tightly into the protein binding pocket of JUN via hydrogen bonds with Ile32 (2.82 Å) and Glu109 (2.59 Å), and hydrophobic interactions with residues Gln117, Asn114, Ile86, Met108, Ala53, and Leu168. Lastly, isovitexin was also predicted to dock tightly into the protein binding pocket of CHEK1 via hydrogen bonds with Tyr20 (3.24 Å, 3.16 Å), Lys132 (3.09 Å), and Cys87 (3.04 Å, 3.09 Å), and hydrophobic interactions with residues Glu134, Asp148, Gly18, Lys38, Ala36, Val23, Glu85, Tyr86, Leu15, Leu137, and Glu91. The results of molecular docking are shown in Figure 7. The docking pattern for each protein and its components is divided into three subplots: *a*, *b*, and *c*. In our schema, “a” shows the binding position of the small-molecule compound (sticks model) on the associated protein (cartoon model) as a whole; “b” shows the interaction between the small molecule (green) and the key residue (grey) on the protein of interest, which facilitates the observation of the spatial stacking between the compound and the protein; and “c” shows a two-dimensional view of the hydrogen bonding and hydrophobic (stacking) interactions between the compound and protein residues.

### 3.7. Inhibition of Isovitexin on KG1-A and HL-60 Cells

**Figure 8:**
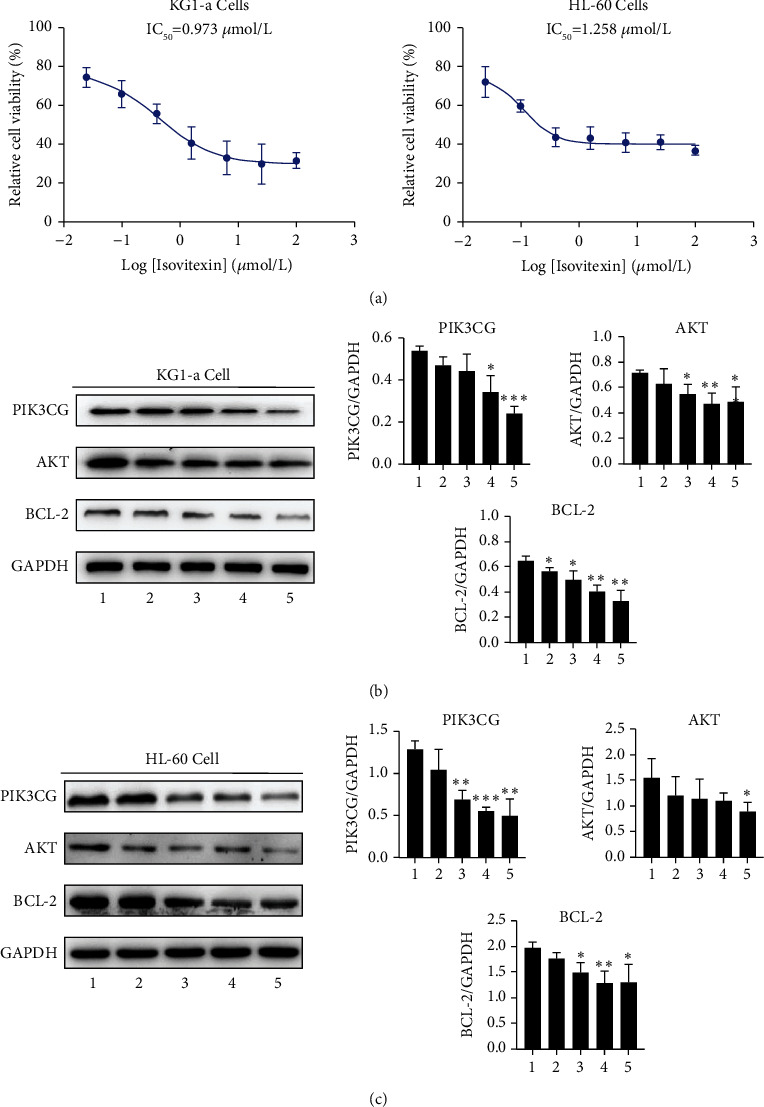
Experimental verification against AML activity of isovitexin in vitro. (a) Isovitexin inhibits the growth of KG1-a cells and HL-60 cells. (b) In KG1-a cell, isovitexin regulated the PI3K-AKT signaling pathway and reduced the expression of Bcl-2. (c) In HL-60 cell, isovitexin regulated the PI3K-Akt signaling pathway and reduced the expression of Bcl-2 in a dose-dependent manner. 1: control group; 2 : 0.39 *μ*mol/L; 3 : 1.56 *μ*mol/L; 4 : 6.25 *μ*mol/L; 5 : 25 *μ*mol/L. GAPDH was used as an internal loading control.

In comparison to the control group, different concentrations of isovitexin had inhibitory effects on KG1-a cells and HL-60 cells in a dose-dependent manner. As shown in Figure 8(a), the isovitexin' IC50 was found to be 0.973 *μ*mol/L (KG1-a cells) and 1.258 *μ*mol/L (HL-60 cells). These results suggest that isovitexin can inhibit AML cell growth.

### 3.8. Isovitexin Regulated the PI3K-AKT Signaling Pathway in AML Cells

Based on the “3.5.1 GO and KEGG” pathways analyzed and “3.6 Molecular Docking” docking results, the PI3K-AKT signaling pathway was selected for experimental validation as a major tumor-related signaling pathway. As shown in Figure 8(b), 8(c), after 48 h treatment with different concentrations of isovitexin (0.39, 1.56, 6.25, 25 *μ*mol/L), the protein levels of PIK3CG, AKT, and BCL-2 in KG1-a cells and HL-60 cells were decreased in a dose-dependent manner (^∗^*p* < 0.05, ^∗∗^*p* < 0.01, and ^∗∗∗^*p* < 0.001 compared with the control group). Taken together with the results of cell viability measurement, the anti-acute myeloid leukemia activities of isovitexin seem to be mediated by inhibition of the cell growth and regulation of the PI3K-AKT signaling pathway.

## 4. Discussion

AML is a highly complex cancer in terms of its molecular and cytogenetic architecture, which are involved with multiple genes or signaling pathways during the development and progression of the disease [32]. Because TCM herbal formulas are composed of multiple compounds, they are generally assumed to work through “multitarget and multipath effects”. Previous research has shown that formulas generally have synergistic effects that may regulate multiple biological processes and pathway networks in the body [8, 33]. However, the characteristic of TCM makes in-depth research on the underlying mechanisms quite challenging. Accordingly, there is an urgent need for new approaches to systematically and comprehensively study the mechanisms of actions of single and compound medicinal substances used in clinical practice in TCM. With the rapid development of systems biology and silicon technologies, network pharmacology and molecular docking have become an emerging approach to clarifying the molecular and underlying pharmacological mechanisms of TCM [34, 35]. In the current study, our team used this approach to systematically study the pharmacological mechanisms by which QHP can alleviate AML.

Some compounds did not exhibit pharmacokinetic properties that could allow them to be delivered to target organs to generate biological activities. In modern integrated drug development, these compounds with OB ≥ 30% and DL ≥ 0.18 were seen as good pharmacokinetically active compounds [36]. There is no relevant data on As_2_O_3_ and As_4_S_4_ that could be retrieved from the TCMSP database including OB and DL values. However, there is increasing evidence to associate both As_2_O_3_ and As_4_S_4_ with the AML pathological process [37–39]. Moreover, it is clear that As_4_S_4_ and As_2_O_3_ are the primary bioactive compounds in QHP [40]. In this study, isovitexin was the most significantly active compound, followed by qingdainone, isoindigo, indigo, indirubin, and quindoline. Isovitexin was shown to have anti-inflammatory and antitumor effects via inhibiting the production of nitric oxide, manganese superoxide dismutase, and ameliorating the bleeding [41, 42]. More specifically, the antileukemia actions of isovitexin, isoindigo, and indirubin were confirmed by in vitro and in vivo models, which enhanced G1/*G*0 arrest and PML-RAR*α* degradation [43, 44]. Meanwhile, our experimental studies also implicated that isovitexin significantly inhibits the proliferation of KG1-a cells and HL-60 cells. But, we still need to conduct further studies on the antileukemia effects of QHP.

In the process of anticancer drug discovery, the search for key targets that are linked together is a major focus of research. One drug can be linked to many genes and proteins in complex relationships. Similarly, one gene or protein can be linked to many drugs. At the same time, there are more and more online analysis tools that can be used in network pharmacology studies. In this study, 89 potential target genes related to QHP and AML were identified with TCMSP, TCMID, SymMap, OMIM, GeneCards, DisGenet, and Drugbank database analysis. After that, 89 potential target genes were analyzed based on the STRING database. In order to comprehensively understand their biofunctional annotation information, GO terms, and KEGG pathway enrichments, they were analyzed with R language. According to the analysis of BP terms, QHP is closely related to aging, peptidyl-serine modification, peptidyl-serine phosphorylation, protein autophosphorylation, reactive oxygen species metabolic processes, and so on. These results may be related to targeting AML effects of QHP [45, 46]. What's more, 5 of the first 20 enriched KEGG pathways are related to PI3K-Akt signaling pathway, Epstein-Barr virus infection, Kaposi sarcoma-associated herpesvirus infection, proteoglycans in cancer, and EGFR tyrosine kinase inhibitor resistance. These results suggest that QHP may have antiviral and anticancer effects and that the relevant pathway is the PI3K-Akt signaling pathway.

In addition, we made a C-T-P network diagram. In this network, nodes with high-degree may be considered to represent the major therapeutic effects of QHP on AML. Compared to macromolecular compounds, the molecular weights of As_4_S_4_ and As_2_O_3_ are small. Despite their high degree, the binding effects of As_4_S_4_ and As_2_O_3_ with proteins are not as strong as that of macromolecules. Moreover, it is significant that these target genes with high-degree are closely related to the PI3K-Akt signaling pathway. Biological studies showed that the dysregulation of the PI3K-Akt signaling pathway in AML may cause the activation of downstream signal molecules, regulate tumor cell proliferation, apoptosis processes, and mediate tumor cell resistance to radiotherapy and chemotherapy [47, 48]. Dysregulation of the PI3K-Akt pathway in AML has become the focus of drug research and development. Thus, targeting the PI3K-Akt pathway in AML may be a potential therapeutic approach [49, 50]. Simultaneously, the molecular docking results showed that the PIK3CG, JUN, and CHEK1 proteins had a good binding affinity with isovitexin. Combined with the results of molecular docking analysis, our western blot analysis results clearly demonstrated that the anti-AML effects of isovitexin were exerted mainly via regulating the PI3K-Akt pathway.

In this study, network pharmacology techniques were used to elucidate the potential antileukemia effects of QHP, and these effects were visualized by molecular docking for verification. This systematic theoretical research provides a fundamental methodology for future pharmacological research designed to further explore the mechanism of action of QHP in the treatment of AML. However, this study has some shortcomings that require further research. Firstly, the public web database selected for this study is characterized by real-time updates, so the results of this study can only partially elucidate the molecular mechanism of QHP against AML. Secondly, only molecular docking and in vitro experimental validation were performed in this study, and a multidimensional validation should be performed subsequently in combination with animal models and clinical trials. Thirdly, studies on the quantitative determination of the content of 14 compounds in QHP are currently not yet perfect. Moreover, isovitexin, though determined as the mainly important bioactive compound of QHP against AML, could not completely stand for QHP. Thus, additional study is required to further explore the underlying molecular mechanism of QHP in the treatment of AML in vitro and in vivo.

## 5. Conclusion

Based on the network pharmacology, molecular docking analysis, and experimental validation, the underlying mechanism of QHP in AML therapy involves the regulation of signaling pathways and targets. In this study, we identified that isovitexin might be the underlying compounds responsible for the therapeutic actions of QHP. The anti-AML effects of QHP seem to be mediated by inhibition of the AML cells growth and Bcl-2 protein expression, and regulation of the PI3K-Akt signaling pathway. Overall, the obtained results suggested that QHP might be used as a promising therapeutic agent for AML and provide additional evidence for the promotion of the wide use of QHP in the clinic for the treatment of AML diseases.

## Figures and Tables

**Table 1:** Description of active compounds of QHP.

Number	Molecule	OB (%)	DL	Molecular structure	Herb
MOL011100	Bisindigotin	41.66	0.39	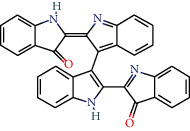	Indigo Naturalis
MOL011105	Indican	34.9	0.23	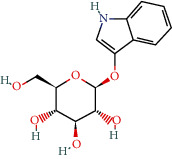	Indigo Naturalis
MOL011332	Quindoline	54.57	0.22	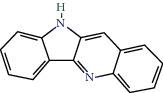	Indigo Naturalis
MOL011335	Isoindigo	94.3	0.26	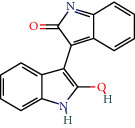	Indigo Naturalis
MOL001753	5-O-Methylvisamminol	37.99	0.25	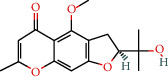	Indigo Naturalis
MOL001781	Indigo	38.2	0.26	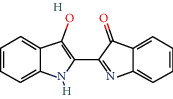	Indigo Naturalis
MOL001810	Qingdainone	45.28	0.89	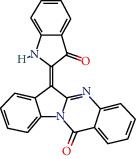	Indigo Naturalis
MOL002309	Indirubin	48.59	0.26	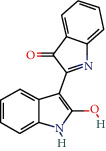	Indigo Naturalis
MOL000358	Beta-sitosterol	36.91	0.75	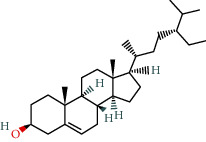	Indigo Naturalis
MOL002322	Isovitexin	31.29	0.72	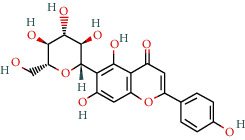	Indigo Naturalis
N/A	As_2_O_3_	N/A	N/A	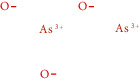	Realgar
N/A	As	N/A	N/A	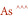	Realgar
N/A	AsS	N/A	N/A	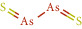	Realgar
N/A	As_4_S_4_	N/A	N/A	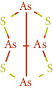	Realgar

**Table 2:** Topological analysis of QHP-AML target network.

Protein name	Gene name	ASPL	BC	CC1	CC2	Degree
Cellular tumor antigen p53	TP53	1.114	0.075	0.898	0.463	78
RAC-alpha serine/threonine-protein kinase	AKT1	1.159	0.046	0.863	0.496	74
Caspase-3	CASP3	1.250	0.021	0.800	0.576	67
Vascular endothelial growth factor A	VEGFA	1.250	0.020	0.800	0.594	66
Epidermal growth factor receptor	EGFR	1.250	0.029	0.800	0.540	66
Interleukin-6	IL6	1.261	0.024	0.793	0.578	65
Tumor necrosis factor	TNF	1.261	0.023	0.793	0.572	65
Transcription factor AP-1	JUN	1.284	0.020	0.779	0.599	64
Catenin beta-1	CTNNB1	1.295	0.020	0.772	0.594	63

ASPL: average shortest path length; BC: betweenness centrality; CC1: closeness centrality; CC2: clustering coefficient.

## Data Availability

Data will be obtained from the corresponding author upon reasonable request.
